# PARP1, DIDO3, and DHX9 Proteins Mutually Interact in Mouse Fibroblasts, with Effects on DNA Replication Dynamics, Senescence, and Oncogenic Transformation

**DOI:** 10.3390/cells13020159

**Published:** 2024-01-15

**Authors:** Agnes Fütterer, Sara Rodriguez-Acebes, Juan Méndez, Julio Gutiérrez, Carlos Martínez-A

**Affiliations:** 1 Department of Immunology and Oncology, Centro Nacional de Biotecnología (CNB-CSIC), 28049 Madrid, Spain; futterer@cnb.csic.es; 2 DNA Replication Group, Molecular Oncology Programme, Spanish National Cancer Research Center (CNIO), 28029 Madrid, Spain; sarodriguez@cnio.es (S.R.-A.); jmendez@cnio.es (J.M.)

**Keywords:** *Dido1* gene, DIDO3, PARP1, DHX9, replication fork, replication stress, senescence, oncogenic transformation

## Abstract

The regulated formation and resolution of R-loops is a natural process in physiological gene expression. Defects in R-loop metabolism can lead to DNA replication stress, which is associated with a variety of diseases and, ultimately, with cancer. The proteins PARP1, DIDO3, and DHX9 are important players in R-loop regulation. We previously described the interaction between DIDO3 and DHX9. Here, we show that, in mouse embryonic fibroblasts, the three proteins are physically linked and dependent on PARP1 activity. The C-terminal truncation of DIDO3 leads to the impairment of this interaction; concomitantly, the cells show increased replication stress and senescence. DIDO3 truncation also renders the cells partially resistant to in vitro oncogenic transformation, an effect that can be reversed by immortalization. We propose that PARP1, DIDO3, and DHX9 proteins form a ternary complex that regulates R-loop metabolism, preventing DNA replication stress and subsequent senescence.

## 1. Introduction

DNA replication stress, i.e., the slowdown and collapse of replication forks, can result in DNA double-strand breaks and thereby promote genomic instability, a hallmark of cancer development. Several pathways and factors able to cause replication stress have been identified, among them altered R-loop dynamics [1,2,3]. R-loops are RNA/DNA hybrid structures present in cell nuclei as a normal consequence of transcription. These structures have physiological roles in regulating gene expression, but when accumulated at high levels can interfere with normal DNA metabolism [4,5]. R-loops are prone to single-strand DNA breaks and are sites of DNA replication fork stalling [6,7]. Both of these phenomena can commit cells to death or senescence [8].

Cells have mechanisms to resolve excess R-loops, among which, topoisomerases and helicases have central roles [6,9]. DHX9 is an RNA helicase that has been directly implicated in R-loop regulation [10,11]. PARP1 is also related to R-loops and the maintenance of genomic stability [10,12]. In mammals, the poly-ADP-ribosylation (PARylation) of different protein substrates is carried out by the poly (ADP-ribose) polymerase (PARP) family of enzymes, the most abundant of which is PARP1 [13,14]. PARP1 pleiotropic functions are implicated in DNA repair, inflammation, and cancer, as well as in mRNA metabolism [15,16]. PARP1 activity is also important for the efficient repair of DNA damage arising at misregulated R-loops [17,18].

The gene *Dido1* encodes three protein isoforms: DIDO1, DIDO2, and DIDO3. The three isoforms share the DIDO1 amino acid sequence, whereas DIDO2 and DIDO3 have progressively extended C-terminal ends. Both the *Dido1* gene structure as well as DIDO protein domain arrangement across DIDO3 are well conserved between mouse and human [19]. DIDO3 is the best known isoform; it is a ubiquitously expressed multifunctional protein implicated as a reader in chromatin modification [20] and mRNA metabolism [21]. It was also shown to interact with DHX9 in the context of R-loops, which accumulate in mutant cells that are deficient in DIDO3 function [22]. In primary mouse embryonic fibroblasts (MEF), this mutation leads to diminished in vitro reprogramming capacity, whereas immortalization not only restores this capacity, but exacerbates it [22].

Here, we show that PARP1 interacts with DHX9 and DIDO3 in mouse cells, and DIDO3 truncation impairs the interaction between PARP1 and DHX9. Concomitantly, DIDO3-deficient primary MEF exhibit stressed DNA replication forks, as well as a senescence phenotype. These MEF also show reduced in vitro oncogenic transformation which, in line with the reprogramming results, is reversed by immortalization.

Based on these data, we propose that the DIDO3 protein acts as a scaffold for recruiting DHX9 and PARP1 to sites in the chromatin where these proteins help resolve R-loops and repair DNA breaks. DIDO3 would thereby prevent DNA replication stress and subsequent senescence, with an impact on cell susceptibility to oncogenic transformation.

## 2. Materials and Methods

### 2.1. Cell Culture

The production of primary MEF bearing the *Dido1^tm3Cmar^* conditional mutation (a floxed exon 16) has been reported, as well as its recombination to actual deletion *Dido1^tm3.1Cmar^* (DIDO3 C-terminal truncation; dE16) by infecting cells with a Cre-expressing adenovirus (Ad5CMV-Cre from Viral Vector Core Facility, University of Iowa, Iowa City, IA, USA) [22]. The immortalization of *Dido1^tm3Cmar^* MEF using HPV E6 + E7 oncogenes and the derivation of *Dido1^tm3.1Cmar^* immortalized MEF clones has also been described [21]. All experiments involving MEF were performed in biological triplicates (independent MEF stocks were prepared from different embryos), except where otherwise indicated. NIH-3T3 cells were obtained from the ATCC repository. Primary MEF were cultured in DMEM High Glucose (Biowest, Nuaillé, France) supplemented with L-glutamine, Na-pyruvate, penicillin/streptomycin, 10% fetal calf serum, and 2-mercaptoethanol. Immortalized MEF were cultured in the same medium without 2-mercaptoethanol.

Stocks of PARP1 inhibitor Olaparib (AZD2281; Selleckchem, Houston, TX, USA) and Topoisomerase1 inhibitor Camptothecin (Sigma-Aldrich, Merck, Darmstadt, Germany) were prepared at 1 mM in DMSO and added to cell cultures at indicated concentrations and times.

Transfection experiments were performed on NIH-3T3 cells with expression plasmids based on pCAGG, and Lipofectamine 2000 reagent (Invitrogen, Thermo Fisher, Waltham, MA, USA) in Opti-MEM (Gibco, Thermo Fisher, Waltham, MA, USA), according to manufacturer’s protocol.

### 2.2. Immunofluorescence

Cells were fixed with ice-cold methanol, permeabilized with 0.1% Triton X-100, blocked with 5% BSA/PBST and stained with the primary antibody for up to 2 h at room temperature (RT). Secondary antibodies were applied (1 h, RT); after washing, slides were mounted with DAPI-containing media.

Images were obtained using a Zeiss laser scanning confocal microscope and processed with Fiji [23]. The Pearson correlation coefficient was calculated with the JACoP plugin [24].

### 2.3. Immunoprecipitation and Western Blotting

Cells were lysed in Cell Lysis Buffer (Cell Signaling, Danvers, MA, USA) supplemented with 1 mM PMSF, incubated with the indicated antibody (4 h, 4 °C) and precipitated with Protein A- or G-loaded magnetic beads (Dynabeads, Thermo Fisher, Waltham, MA, USA). After extensive washing, immunoprecipitates were released in a denaturing electrophoresis sample buffer, separated in SDS-PAGE, and transferred to nitrocellulose membrane (Bio-Rad, Hercules, California, USA). The membrane was blocked with 5% milk powder in TBST and probed with indicated primary antibodies (overnight, 4 °C). The following day, membranes were incubated with a horseradish peroxidase-coupled secondary antibody and developed for luminescence using Western Lightning Plus-ECL (PerkinElmer, Shelton, CT, USA).

Af1521 Macrodomain Magnetic Resin (Tulip Biolabs, Lansdale, PA, USA) was used to precipitate mono- and poly-ADP ribosylated proteins [25], according to the manufacturer’s protocol.

### 2.4. Stretched DNA Fiber Assays

To analyze DNA replication fork rate, cells were sequentially pulse-labeled with CldU (20 min) and IdU (20 min). For fork symmetry analysis, cells were sequentially pulse-labeled with CldU (10 min) and IdU (30 min). In all cases, DNA fibers were spread in a buffer containing 0.5% SDS, 200 mM Tris pH 7.4, and 50 mM EDTA. CldU and IdU tracks were detected using immunofluorescence with rat anti-BrdU antibody for CldU (Abcam, Cambridge, UK, ab6326) and mouse anti-BrdU for IdU (BD Biosciences, Franklin Lakes, NJ, USA, 347580). DNA fiber integrity was assessed with a mouse anti-ssDNA antibody (Sigma-Aldrich, Merck, Darmstadt, Germany MAB3034). Stretched DNA fiber images were obtained with a Leica DM6000 B microscope, as described in [26]. The conversion factor used was 1 µm = 2.59 kb [27]. Signals were measured and quantified using ImageJ software [28]. For fork rate assessment, 250–350 forks (red-green tracks) were measured per condition in each independent experiment. For replication origin symmetry assessment, both green tracks stemming from a single origin were measured (*n* = 100 origins per condition in each independent experiment). An origin was considered asymmetric when the ratio between the two forks (long/short) was >1.4.

### 2.5. Senescence-Associated β-Galactosidase Staining

The in situ labeling of primary MEF was carried out using the Senescence beta-Galactosidase Staining Kit (Cell Signaling, Danvers, MA, USA), according to manufacturer’s instructions. For flow cytometry measurements, the CellEvent Senescence Green Assay Kit (Invitrogen, Thermo Fisher, Waltham, MA, USA) and a Cytomics FC-500 cytometer were used.

### 2.6. In Vitro Oncogenic Transformation and Colony-Forming Assays

To test the susceptibility of primary MEF to oncogenic transformation, we first produced a recombinant retrovirus encoding E1A and H-RasV12 oncogenes. In brief, we transfected host HEK-293 cells with pPLC-E1A+Ras or empty pPLC plasmids; 72 h later, the retrovirus-containing or control supernatants were harvested, filtered, and used to infect the target MEF. Approximately 15 days after infection, plates were stained with Giemsa (Sigma-Aldrich, Merck, Darmstadt, Germany) to visualize upcoming transformed MEF foci.

E6+E7-immortalized MEF were seeded at 1000 cells/10 cm plate and cultured without further additions; after 10 days, their clonogenic survival ability was monitored by staining plates with 2% methylene blue in 50% ethanol and counting arising colonies.

### 2.7. Antibodies

Primary antibodies (Ab): anti-DHX9 rabbit monoclonal Ab (Invitrogen, ThermoFisher, Waltham, MA, USA; ARC1033; WB 1:1000; IF 1:200), anti-PARP1 rabbit monoclonal Ab (Cell Signaling, Danvers, MA, USA; 46D11; WB 1:1000; IF 1:500), anti-HA-tag mouse monoclonal Ab (BioLegend, San Diego, CA, USA; MMS-101P; WB 1:1000), anti-HA-tag rabbit polyclonal Ab (Abcam, Cambridge, UK; ab9110; WB 1:4000); anti-EZRIN rabbit polyclonal Ab (Cell Signaling, Danvers, MA, USA; WB 1:1000). Anti-NT-DIDO mouse monoclonal Ab (MAB-1C6; IF 1:10) and anti-DIDO3-CT-specific rabbit polyclonal Ab (PAB-DIDO3) were custom-produced by the Protein Tools unit in Centro Nacional de Biotecnología (Madrid, Spain).

For immunoprecipitation experiments, 3–5 μg were used per sample.

## 3. Results and Discussion

### 3.1. PARP1 and DHX9 Interact in Mouse Embryonic Fibroblasts

**Figure 1:**
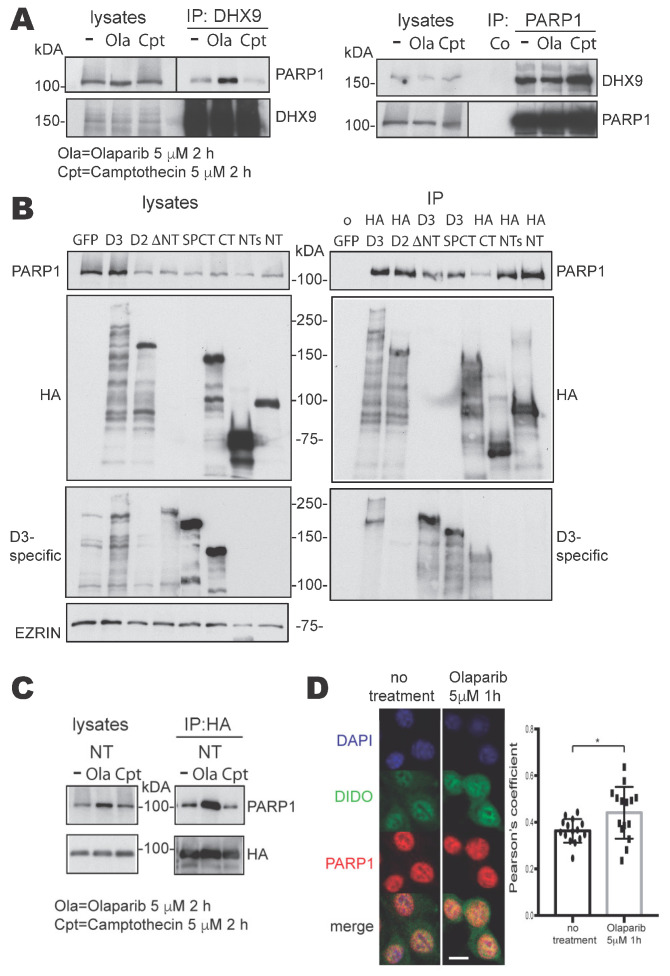
Interactions between PARP1 and DHX9 or DIDO3, and the mapping of binding domains. (**A**) Western blots of untreated (−), Olaparib (Ola)- or Camptothecin (Cpt)-treated NIH-3T3 cell lysates and their immunoprecipitates. Immunoprecipitation was performed with anti-DHX9 antibody (left) or anti-PARP1 antibody (right). The proteins probed on each blot are labeled on their sides. (Note: Olaparib treatment reduces the PAR load in proteins, which sharpens bands). (**B**) Western blots of samples from transfected NIH-3T3 cells expressing GFP, as a negative control, or different DIDO3 protein domains (See Supplementary Figure S1 for details): lysates (left) and immunoprecipitates (right). Anti-HA antibody was used for immunoprecipitation, except for ∆NT and SPCT samples, in which an anti-DIDO3 CT-specific antibody was used. The blots were probed with anti-PARP1, -HA, -D3-specific antibodies and with anti-EZRIN as loading control. (**C**) Western blots of samples from NIH-3T3 cells transfected with the HA-DIDO-NT construct, untreated (−) or treated with Olaparib or Camptothecin: lysates (left) and immunoprecipitates with anti-HA (right) were probed with anti-PARP1 and -HA antibodies. (**D**) Confocal immunofluorescence images of untreated (left) or Olaparib-treated (right) NIH-3T3 cells stained with anti-DIDO3 (green), anti-PARP1 (red) and DAPI (blue). The co-localization of DIDO3 and PARP1 stains in individual cells was quantified as a Pearson correlation coefficient; the plot shows the correlation coefficients of 15 cells from each treatment group. A two-tailed Student’s *t*-test showed a statistically significant difference in the mean degree of DIDO3 and PARP1 co-localization between untreated and treated cells. * *p* < 0.05.

The human PARP1 and DHX9 proteins were previously shown to interact in the context of RNA/DNA complexes [10,29]. To study the potential interaction of the homologous proteins in mouse cells, lysates of NIH-3T3 MEF were immunoprecipitated (IP) with anti-PARP1 or -DHX9 antibodies. Samples of input lysates and precipitates were Western blotted and probed with criss-crossed anti-DHX9 and -PARP1. Bands corresponding to both proteins were detected in both precipitates (Figure 1A), suggesting that the PARP1 and DHX9 proteins also interact in mouse cells.

The inhibition of PARP1 activity not only abolishes the PARylation of proteins, but also leads to increased R-loop levels [12]; therefore, we investigated whether this inhibition could alter the PARP1-DHX9 interaction. We treated NIH-3T3 cells with Olaparib prior to lysis and IP. While this treatment did not alter the amount of PARP1 protein in the lysate, more PARP1 co-precipitated with DHX9 (Figure 1A). In contrast, treatment with Camptothecin, an inhibitor of Topoisomerase I that also increases R-loops [30], did not stimulate the co-immunoprecipitation (Co-IP) of PARP1 (Figure 1A). These results suggest that the interaction between DHX9 protein and PARP1 inversely depends on the enzymatic activity of the latter.

### 3.2. Study of the Interaction between DIDO3 and PARP1 in Mouse Cells

As the DHX9 protein interacts with the DIDO3 C-terminal region in the context of R-loops [22], we tested whether DIDO3 also interacts with PARP1 and, if so, which domains are involved. To overcome the limited performance of some anti-DIDO3 antibodies in the IP technique, and for ease of mapping the potential binding region, we performed experiments on NIH-3T3 cells transiently transfected with plasmids encoding HA-tagged-DIDO3, -DIDO2 or -DIDO3 parts (Figure S1). Therefore, an anti-HA antibody was used as the main tool for IP. PARP1 was co-immunoprecipitated from lysates of HA-DIDO3-transfected cells (Figure 1B), which suggested that DIDO3 interacts with endogenous PARP1. HA-DIDO2 showed essentially the same result, indicating that the C-terminal half of DIDO3 is mostly dispensable for this interaction. The HA-DIDO3-CT construct further confirmed that PARP1 interacts very poorly with the C-terminal part alone (Figure 1B).

In contrast, the deletion of N-terminal stretches of DIDO3 as short as 423 amino acids notably decreased the amount of PARP1 in the immunoprecipitate (Figure 1B), whereas protein fragments corresponding to the N-terminal 528 (HA-DIDO-NT) or 340 (HA-DIDO-NTs) amino acids of DIDO3 co-immunoprecipitated as much PARP1 as whole DIDO3 (Figure 1B).

These findings are in agreement with recent studies in human cells that reported, respectively, the interaction between PARP1 and a Flag-tagged 529 N-terminal fragment of DIDO1 [31] and the lack of PARP1 interaction with N-terminal truncated DIDO isoforms [32]. Together these results reinforce the concept that the DIDO3 protein binds PARP1 by its N-terminal part, the opposite to DHX9, both in mice and in humans.

Both DIDO3 [22,33] and PARP1 [16,34] are reported to regulate RNA metabolism, and both can bind to chromatin [35,36]. Although our lysis and IP conditions were not favorable to RNA or chromatin preservation, we analyzed whether nucleic acids play a role in the observed DIDO-PARP1 interaction.

To test a potential role for RNA, lysates of HA-NT-DIDO-transfected cells were treated with RNase A before and during HA-IP, and the blot was probed with anti-PARP1 as above. The treatment did not reduce the amount of co-immunoprecipitated PARP1, which suggests that RNA does not mediate the DIDO3-PARP1 interaction (Supplementary Figure S2A). A similar experiment with DNase was inconclusive, as the enzymatic conditions interfered with the IP. To address possible chromatin involvement in the interaction, we used a modified HA-DIDO-NT construct with an inactivating mutation in the PHD domain (amino acids 265–319) that mediates DIDO binding to histone H3 in euchromatin [20]. A plasmid encoding the modified HA-DIDO-NT construct was transfected into NIH-3T3 fibroblasts, which were lysed and immunoprecipitated as above. The capacity of the construct to co-immunoprecipitate PARP1 was identical to the unmodified HA-DIDO-NT- or NTs (Supplementary Figure S2B). This suggests that, as with RNA, chromatin does not mediate the DIDO3-PARP1 interaction, which seems to be direct.

To study whether PARP1 activity influences its interaction with DIDO3, as was the case with DHX9, we treated cells transfected with the HA-DIDO-NT construct with Olaparib or Camptothecin, as described above. The results again showed more PARP1 co-immunoprecipitated in the case of Olaparib but not for Camptothecin (Figure 1C).

To further illustrate the interaction between endogenous, full-length DIDO3 and PARP1 proteins not only in transfected, but also in untransfected NIH-3T3 cells, samples of the latter were untreated or treated with Olaparib, fixed and immunostained with anti-DIDO3 and anti-PARP1 antibodies. Confocal imaging showed a co-localization of endogenous DIDO3 and PARP1 proteins in nuclei; in addition, it showed that Olaparib treatment quantitatively increased the co-localization of these proteins, which fully corroborates the Co-IP results in transfected cells (Figure 1D).

Overall, these results suggest that PARP1 binds the DIDO3 protein N-terminal part, in contrast to DHX9, which binds to the C-terminal domain. The DIDO3-PARP1 interaction is independent of DIDO PHD domain integrity and is not RNA-mediated, but is inversely dependent on PARP1 enzymatic activity, as is the case for the PARP1-DHX9 interaction.

### 3.3. DIDO3 Mediates the Interaction between PARP1 and DHX9

**Figure 2:**
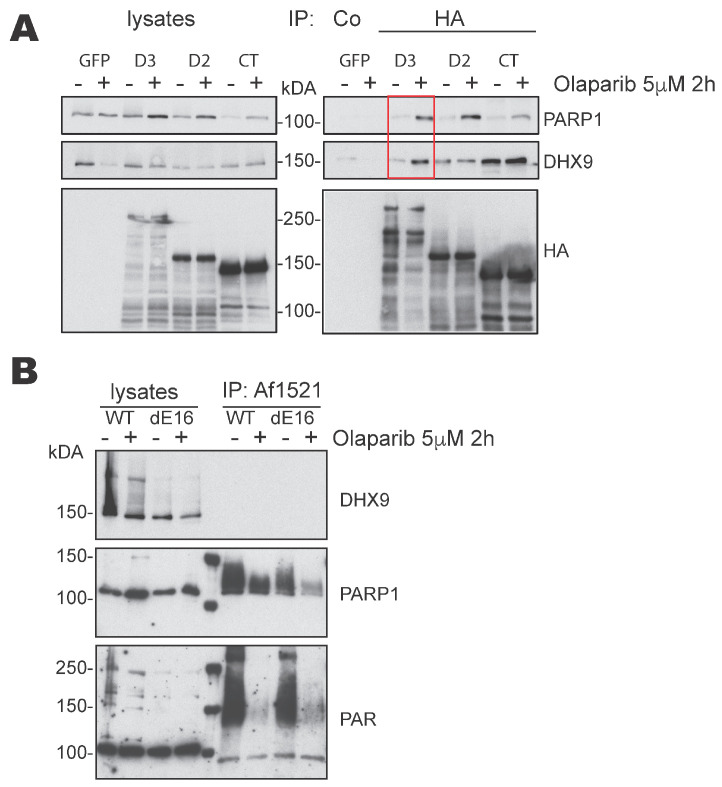
Interaction of DIDO3 with PARP1 and DHX9. (**A**) Western blots of samples from transfected NIH-3T3 cells expressing GFP, as a negative control, or HA-tagged-full-length DIDO3, -DIDO2 or -DIDO3-CT, untreated (−) or treated with Olaparib (+). The cells were lysed (left) and immunoprecipitated with anti-HA antibody (right); the blots were probed for PARP1, DHX9, and HA-tagged proteins. Olaparib treatment increased the co-precipitation of both proteins only in the case of full-length DIDO3 (red box). (**B**) Western blots of WT and DIDO3 mutant (dE16) cell lysates and their precipitates, previously untreated (−) or treated with Olaparib (+). Precipitation was performed with magnetic beads covalently linked to Af1521 Macrodomain, which specifically binds to PARylated proteins. The blots were probed with anti-DHX9 antibody (upper), anti-PARP1 antibody (middle) and anti-PAR antibody (lower panel).

As shown above, in MEF, the DIDO3 protein can act as a physical link between PARP1 and DHX9, binding each of these proteins to each of its ends; in addition, the linkage seems stronger when PARP1 is not self-PARylated. To substantiate this hypothesis, we used HA-DIDO3-, HA-DIDO2-, or HA-DIDO-CT-transfected cells, previously incubated alone or with Olaparib, in IP experiments similar to those described above. The blot was probed with both anti-PARP1 and anti-DHX9 antibodies (Figure 2A). The results show that Olaparib treatment enhanced the simultaneous Co-IP of both PARP1 and DHX9 proteins only in the case of HA-DIDO3 full-length-transfected cells. Olaparib only clearly increased the Co-IP of PARP1 in HA-DIDO2-transfected cells, in accordance with our results above, whereas only DHX9 was intensified in HA-DIDO3-CT cells, as reported in [22].

These results support the idea that, in mouse fibroblasts, the DIDO3 protein mediates PARP1-DHX9 interaction to form a ternary complex, and that this interaction is more efficient in conditions of quiescent PARP1 activity. In human cells, proteomics data also point to molecular proximity among these three proteins at stressed replication forks, which was also increased by PARP1 inhibition [37]; this coincides fully with our results.

These interactions suggested that PARP1 could PARylate DHX9 on the DIDO3 scaffold. The PARylation of helicases is proposed to activate their unwinding activity [12]. We tested whether the DIDO3 C-terminal truncation mutation (dE16), which disables DIDO3 binding to DHX9 [22], affected the hypothetical PARylation of DHX9. For this, we used wild-type (WT) and dE16 MEF, untreated or treated with Olaparib, lysed with RIPA, and precipitated with PAR-binding Af1521 Macrodomain magnetic beads [25]. Lysates and precipitates were Western blotted and the blots probed with anti-DHX9, -PARP1 or -PAR antibodies. We detected the DHX9 protein in all lysate samples, but not in the Af1521 IP samples (Figure 2B, top panel), which suggests that DHX9 is not PARylated in MEF. In contrast, we found abundant smeared PARP1 in Af1521 precipitates from untreated samples, consistent with the idea that PARP1 is itself PARylated [12]. This validates the ability of the Af1521 beads to precipitate a PARylated protein in our workflow. The amount, as well as the smearing of PARP1, was reduced in Olaparib-treated samples, confirming inhibitor function and the accuracy of the analysis (Figure 2B, center). We observed a slightly reduced amount of PARylated PARP1 in dE16 MEF compared to WT samples, which suggests that, although PARP1 binds the DIDO3 N-terminal part, the C-terminal part influences PARP1 binding and possibly PARP1 activity. The blot probed with anti-PAR corroborated that the Af1521 beads precipitated a range of PARylated proteins in addition to PARP1, from both WT and dE16 MEF lysates, further strengthening the finding that the DHX9 protein is not PARylated in MEF.

Therefore, the functional significance of the PARP1-DHX9 interaction is not evident. We speculate that the two proteins, along with DIDO3, remain mutually bound and inactive until they are required to resolve R-loops. PARP1 may then be activated and PARylate itself as well as other substrates, although not DHX9. The bulky, charged PAR groups in PARP1 can weaken its interaction with DHX9, which would more easily unwind RNA from the template DNA strand. A similar mechanism has been proposed for other helicases such as WRN, which is structurally similar to DHX9 [38]; PARP1 also mediates the association of the DDX18 helicase with R-loops, in a way dependent on its auto-PARylation [39].

Although further research is needed to clarify the role and complexity of PARP1-DIDO3-DHX9 interactions, it is clear that the C-terminal truncation of DIDO3 in MEF partially disturbs these interactions, resulting in accumulated R-loops as well as signs of DNA replication stress [22].

### 3.4. DIDO3-Deficient Cells Show Altered DNA Replication Fork Dynamics

**Figure 3:**
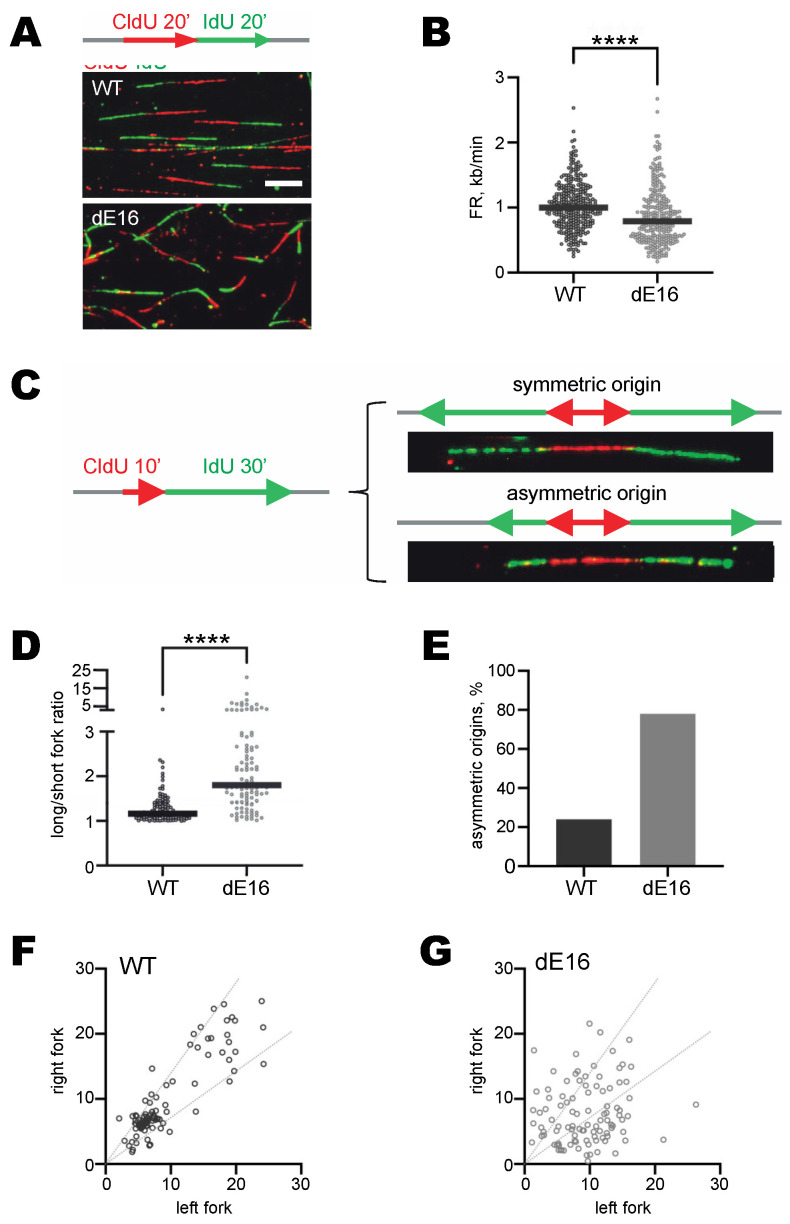
Analysis of DNA replication dynamics in stretched DNA fibers of WT and DIDO3 mutant dE16 MEF. (**A**) Top: schematic of sequential labeling with CldU and IdU for the analysis of fork progression rate. The length of green tracks following red tracks (active forks) is measured. Bottom: representative images of stretched DNA fibers of WT and DIDO3-deficient (dE16) cells. Bar, 10 μm. (**B**) Plot of fork progression rates (*n* > 250 forks per condition). (**C**) Left: labeling schematic for origin asymmetry analysis. Right: representative images of symmetric and asymmetric origins. The length of the two green signals emanating from each origin is measured. (**D**) Representation of the ratio between the long and short fork of each origin (*n* = 100 origins/condition). Thick black lines indicate the median value in each case. (**E**) Histogram shows the percentage of asymmetric origins (long/short ratio greater than 1.4). (**F**,**G**) Individual measures of the length of right and left forks arising from each single origin, as measured in WT (**F**) and DIDO3 mutants dE16 (**G**) cells. Dotted lines represent a deviation of 40% from the bisector of the graph (ideal symmetric forks). Statistical analyses were performed with Mann–Whitney tests, **** *p* < 0.0001. One experiment is shown out of two independent replicas with similar results.

The measurement of DNA synthesis in individual DNA molecules is frequently used to identify possible causes of replicative stress [40]. To better understand DNA replication defects in the DIDO3 C-terminal mutant MEF (dE16), we measured the speed of replication forks (referred to as “fork rate”) and the symmetry at DNA replication origins in stretched DNA fibers. The mutant MEF displayed slower fork progression rates than WT (Figure 3A,B). This effect was accompanied by a greater asymmetry between the bidirectional forks arising from single replication origins (Figure 3C,D), as well as a larger proportion of asymmetric origins (Figure 3E–G).

Overall, these results suggest a higher frequency of replication fork stalling in DIDO3 mutant MEF. This is consistent with the idea that replication forks slow down at unresolved accumulated R-loops [41] and constitutes a plausible mechanism underlying the signs of DNA replication stress reported for the DIDO3 mutation [22].

### 3.5. DIDO3-Deficient Cells Show Increased Senescence and Altered Susceptibility to In Vitro Oncogenic Transformation

**Figure 4:**
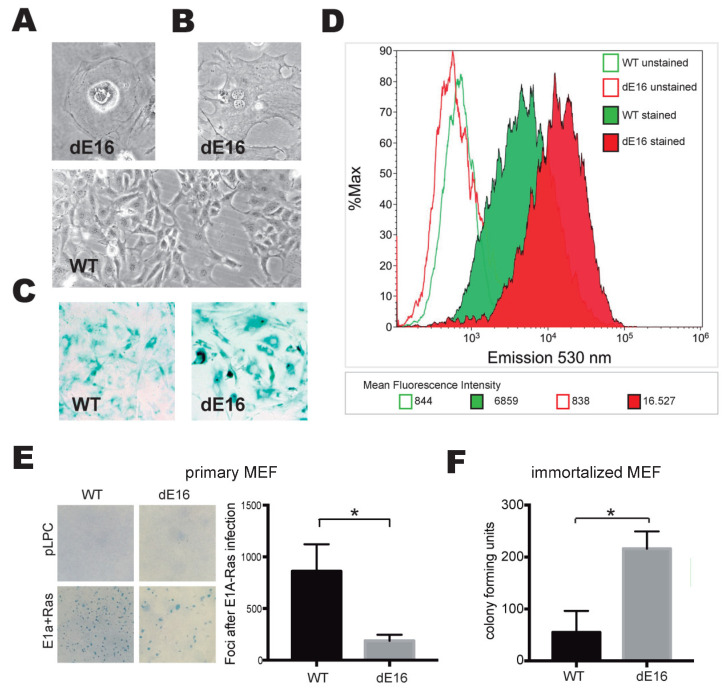
Senescence and oncogenic transformation in DIDO3-deficient MEF. (**A**,**B**) Photographs of cells with morphological features of senescence, frequent in cultures of DIDO3-deficient (dE16) primary MEF. A field of WT MEF at the same optical power is presented below for comparison. (**C**) Representative images of WT vs. DIDO3-deficient MEF stained with X-Gal for senescence-associated β-galactosidase activity. (**D**) Flow cytometry profiles of WT vs. DIDO3-deficient MEF stained with a fluorescent probe sensitive to senescence-associated β-galactosidase activity. Profiles of unstained cells are also shown. The mean fluorescence emission intensity for each population is shown. All results are representative of 3 independent pairs of WT vs. DIDO3-deficient primary MEF. (**E**) Photographs of cellular foci formed after infection with oncogenic retrovirus (E1A+Ras) in WT vs. DIDO3-deficient primary MEF (lower left panel). No foci arose after infection with empty (pPLC) virus (upper left panel). The susceptibility of each MEF genotype to in vitro oncogenic transformation was quantified as foci count per plate (right panel). *n* ≥ 3; bars indicate mean ± SEM. Statistical analysis was performed with two-tailed Student’s *t*-test, * *p* < 0.05. (**F**) Quantification of in vitro cell survival efficiency of WT vs. DIDO3-deficient immortalized MEF as colony count per plate in the clonogenic assay. *n* = 3 for biological samples; in all experiments, at least 3 technical replicates were created for each sample; bars indicate mean ± SEM. Statistical analyses were performed with a two-tailed Student’s *t*-test, * *p* < 0.05.

DNA replication stress can commit cells to senescence [8]. Having documented a higher frequency of fork stalling in DIDO3-deficient cells compared to WT cells, we asked whether this would render them prone to senescence. As early as passages 4–5, cells with a flattened morphology, a spread-out vacuolated cytoplasm, and large nucleus were frequently seen in DIDO3-deficient primary MEF cultures (Figure 4A); in many cases, the cells were binucleated (Figure 4B). All of these features are morphological hallmarks of senescence. Our observations were corroborated by the biochemical in situ staining of senescence-associated β-galactosidase; DIDO3-deficient cells accumulated more stains than WT cells (Figure 4C). For a quantitative measurement of β-galactosidase-positive cells, samples of both cultures were incubated with a fluorescent substrate and analyzed by flow cytometry. The results show a shift to high fluorescence in the DIDO3-deficient cell population compared to WT cells (Figure 4D), confirming the robust expression of senescence-associated β-galactosidase activity in the mutant cells.

We then tested how altered DNA replication dynamics and senescence in DIDO3-deficient cells affect their susceptibility to oncogenic transformation. DIDO3-deficient vs. WT primary MEF were subjected to standard E1A+Ras oncogene-induced transformation assays. The results, measured as focus formation efficiency, showed that DIDO3-deficient primary MEF are less susceptible to transformation than WT cells (Figure 4E). In contrast, E6+E7-immortalized DIDO3-deficient MEF, which do not undergo senescence, showed a significant increase in colony-forming ability in a clonogenic survival assay, compared to WT (Figure 4F).

These results suggest that DIDO3 deficiency in mouse fibroblasts promotes a senescence pathway that protects them from oncogenic insults. If senescence is overcome, however, DIDO3 deficiency causes the opposite effect, promoting uncontrolled cell proliferation. The behavior of the DIDO3-deficient MEF is coherent with that reported in an in vitro reprogramming setting [22], in which primary DIDO3-deficient MEF fail to undergo dedifferentiation and reprogramming to a pluripotent state, but they perform even better than WT when previously immortalized with viral E6+E7 genes. This suggests that p53 and Rb, the senescence triggers targeted by the E6+E7 duo [42], exert efficient opposition to the oncogenic as well as the dedifferentiating biological forces that the DIDO3 deficiency primarily unleash. This is also consistent with the current vision of cancer initiation and cell dedifferentiation as closely related biological mechanisms [43].

## 4. Conclusions

PARP1 [44,45,46] and DHX9 [47,48] are key players in genomic stability and cancer. The present study demonstrates that PARP1 and DHX9 proteins interact in mouse fibroblasts and that DIDO3 facilitates this interaction by acting as a scaffold. The truncation of DIDO3 alters these interactions, with consequences for DNA replication dynamics, senescence, and oncogenic transformation.

**Figure 5:**
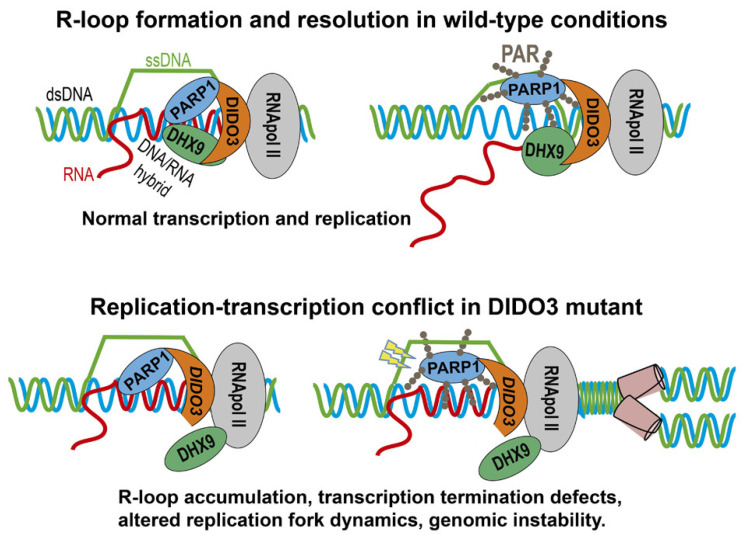
Model for physiological and pathological R-loop formation and resolution. Involvement of DIDO3, PARP1, and DHX9 in the succesful or failed resolution of R-loops in wild-type and DIDO3 CT-truncated conditions, respectively. The respective physiological and pathological consequences are listed.

Based on the present results and previous research [22], we present a molecular model (illustrated in Figure 5): in physiological conditions, PARP1, DIDO3, and DHX9 proteins interact mutually and with R-loops, collaborating in their resolution. In this process, PARP1 becomes enzymatically activated and PARylates itself, resulting in the release of DHX9 and of its RNA-unwinding activity. DIDO3 truncation disturbs these interactions in a manner that results in the pathological accumulation of R-loops and subsequent breaks in the exposed ssDNA. This hampers progression of DNA replication, which stalls replication forks and eventually triggers senescence. In these conditions, oncogenic transformation as well as similar processes such as cell dedifferentiation and reprogramming, become impaired.

Our results could be of interest in the human clinical setting. p53 function, and hence senescence potential, is known to be compromised in more than half of cancers [49]. In these cases, an additional deficiency in DIDO3 function would enhance the tumor proliferative potential, adding further severity to the disease; this would call for more aggressive therapy. In p53-competent tumors, however, DIDO3 deficiency would be able to trigger tumor senescence, or could render the tumor more responsive to therapies focused on this process; DIDO3 deficiency would thus indicate a better prognosis for this patient cohort. Further research will be needed to elucidate the role of DIDO in cancer initiation and progression, and to explore the therapeutic potential of interfering with its function.

## Data Availability

Data are available upon request.

## Supplementary Materials

The following supporting information can be downloaded at: https://www.mdpi.com/article/10.3390/cells13020159/s1, Figure S1: Scheme of full-length DIDO3, DIDO2 and differently truncated parts of DIDO3 proteins encoded in expression plasmids; Figure S2: The DIDO3-PARP1 interaction is independent of RNA and of the integrity of DIDO’s PHD domain.
