# Supplementary material for: PARP1, DIDO3, and DHX9 Proteins Mutually Interact in Mouse Fibroblasts, with Effects on DNA Replication Dynamics, Senescence, and Oncogenic Transformation
Source: Cells. 2024 Jan 15;13(2):159. doi: 10.3390/cells13020159 (PMC10814579; doi:10.3390/cells13020159)
Supplement: Supplementary file 1 [file cells-13-00159-s001.zip › cells-2805424-supplementary.pdf]

Supplementary Figures and Legends

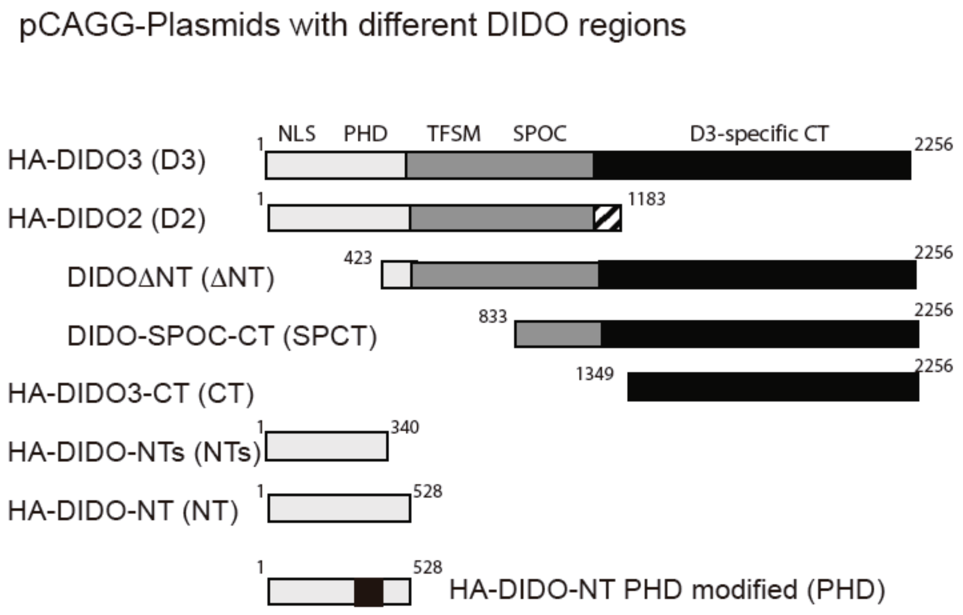

Supplementary Figure S1. Scheme of full-length DIDO3, DIDO2 and differently truncated parts of DIDO3 proteins encoded in expression plasmids. HA denotes amino terminal hemagglutinin-tagging of some of the constructs. Light grey bars denote the common N-terminal part of all three isoforms DIDO1, DIDO2 and DIDO3. The heavy grey central part is common in DIDO2 and DIDO3 isoforms. The filled black C-terminal bar denotes the DIDO3-specific part. The approximate location of canonical protein domains is depicted above the DIDO3 scheme. All constructs were cloned in the plasmid vector pCAGG. The abbreviated name of each construct is written in brackets.

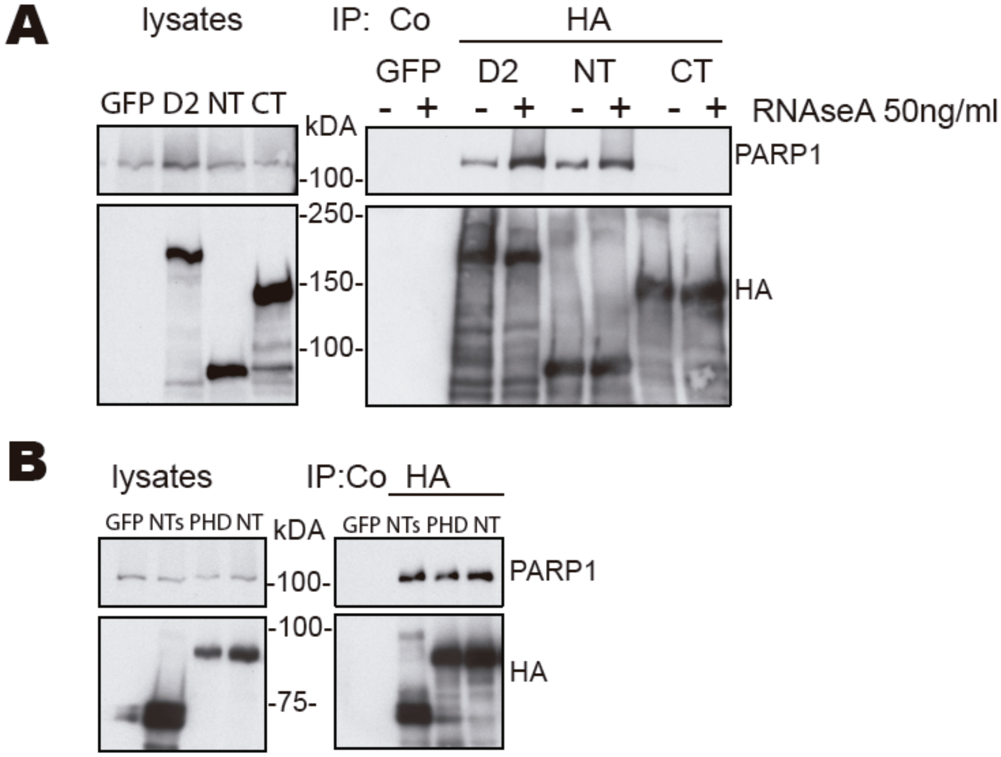

Supplementary Figure S2. The DIDO3-PARP1 interaction is independent of RNA and of the integrity of DIDO's PHD domain. A. Western blots of samples of lysates (left panel) and HA-immunoprecipitates (right panel) of NIH-3T3 transfected cells expressing HA-tagged-DIDO2, -DIDO-NT or -DIDO3-CT, that were untreated (-) or treated (+) with RNAse A along the immunoprecipitation procedure. **B.** Western blots of lysates (left panel) and HA-immunoprecipitates (right panel) of NIH-3T3 transfected cells expressing GFP, as a negative control, or HA-DIDO-NT, HA-DIDO-NTs (a shorter version of the former), or HA-DIDO-NT PHD (a version with an inactivated PHD domain). The 3 constructs encompass the PARP1-binding domain of DIDO3. The proteins probed on each blot are labeled on the right side.
